# The Panorama of Cerebral Palsy in Sweden Part XIV Shows Further Decrease in Prevalence and Severity in the Birth‐Years 2015–2018

**DOI:** 10.1111/apa.70615

**Published:** 2026-05-29

**Authors:** Kate Himmelmann, Magnus Påhlman

**Affiliations:** ^1^ Department of Pediatrics, Institute of Clinical Sciences Sahlgrenska Academy at the University of Gothenburg Göteborg Sweden

**Keywords:** aetiology, cerebral palsy, epidemiology, gestational age, prevalence

## Abstract

**Aim:**

To describe prevalence, background and gross motor function of cerebral palsy (CP) in western Sweden 2015–2018 and compare to birth‐years 1999–2014.

**Methods:**

Population‐based study covering 108 963 live births in 2015–2018. Birth characteristics, neuroimaging findings and motor outcome were analysed and prevalence calculated and compared to previous cohorts.

**Results:**

CP was diagnosed in 194 children, 180 had pre‐ or peri/neonatal background. Crude prevalence was 1.78 per 1000 live births compared to 2.18 in 1999–2002. Gestational age‐specific prevalence for < 28 gestational weeks was 65.9 per 1000 live births, 41.4 for 28–31 weeks, 5.6 for 32–36 weeks and 1.06 per 1000 for > 36 weeks of gestation. Hemiplegia was found in 51.7%, diplegia in 30.6% and tetraplegia in 5.6%, dyskinetic CP accounted for 10.6% and ataxia for 1.7%. Neuroimaging showed maldevelopments in 7%, white matter lesions in 54% and grey matter lesions in 29%. Motor function had improved since the birth years 1999–2002, both in term and in extremely preterm children. Prenatal aetiology was considered in 34.4% and peri/neonatal in 49.4%. Aetiological period remained unclassified in 16.1%.

**Conclusion:**

The prevalence of CP is declining. Gross motor function has improved over time. White matter lesions are increasingly common.

## Summary

- During the time period 1999–2018 the prevalence of cerebral palsy has decreased in western Sweden from 2.18 in the birth‐years 1999–2002 to 1.78 per 1000 live births in 2015–2018.
- The proportion of white matter lesions, the most common neuroimaging finding in children with cerebral palsy, has increased from 1999–2002 to the cohort 2015–2018.
- Gross motor function has improved from 1999–2002 to 2015–2018 in term born as well as in children born extremely preterm.

**Abbreviations**
ACOG: American College of Obstetricians and Gynecologists
CP: cerebral palsy
HIE: hypoxic‐ischaemic encephalopathy
LGA: large for gestational age
MRI: magnetic resonance imaging
SCPE: Surveillance of Cerebral Palsy in Europe
SD: standard deviation
SGA: small for gestational age

## 1 Introduction

Cerebral palsy (CP) remains a global indicator of child health [1]. A decreasing prevalence has been reported in high‐income countries, while the estimated occurrence is higher in low‐ and middle‐income countries [2]. The risk factor spectrum and impairment profile including severity may vary over time [1, 3], and the reasons for this must be sought.

The epidemiology of CP in western Sweden has been monitored since the mid‐1950s by the CP register of western Sweden currently comprising 2703 individuals with CP, born 1954–2018. For the birth‐years 2011–2014, we reported a declining prevalence [3, 4].

The aim of this study was to report the prevalence and background of CP, and trends in gestational age and motor function in children born 2015–2018 compared to the previous birth‐year cohorts 1999–2014 in the study area.

## 2 Patients and Methods

The study area comprised the counties of Västra Götaland, Jönköping and Halland, with a total population of about 2.5 million inhabitants, with a slightly positive net migration [5]. This long‐time study, which has presented prevalence of CP in the study area from 1954 to 2014, now focused on the birth‐years 2015–2018, during which 108 963 live births were recorded, 273 before 28 completed weeks of gestation (extremely preterm), 603 at 28–31 weeks (very preterm), 5015 at 32–36 weeks (moderately preterm) and 94 462 at more than 36 weeks of gestation (born at term). In 8610 cases (8.2% of the latter group), birth occurred after 42 completed weeks (post‐term).

Children with CP were included if they were born in Sweden and lived in the study area on 31 December 2022. All the children had a diagnosis of CP verified at 4–8 years of age by the local neuropaediatrician. In doubtful cases, a second diagnostic assessment was performed by the first author (K.H.). Children diagnosed with CP who had died after 2 years of age were also included.

CP was defined according to an international consensus meeting in Bethesda [6]. The Swedish and internationally accepted classification of CP syndromes was applied [4, 7], in parallel with the CP classification of the Surveillance of Cerebral Palsy in Europe (SCPE) [8], where hemiplegia corresponds to unilateral spastic CP and diplegia and tetraplegia are combined to comprise bilateral spastic CP. The term prenatal referred to the period of pregnancy until the onset of labour resulting in delivery, perinatal to the period from the onset of labour until the seventh day of life, neonatal to the period up to day 28 and post‐neonatal to the period from day 29 to 2 years of age. Maternal disorder was considered in the case of chronic disorder (such as diabetes mellitus, essential hypertension, hypothyroidism, nephropathy, inflammatory bowel disease), pyelonephritis or acute severe illness during pregnancy, or fever of > 38.5°C at delivery [9]. Assisted reproductive technology (ART) was documented. Small for gestational age (SGA) was defined as a birth weight for gestational age of −2SD or less, while large for gestational age (LGA) was defined as more than 2SD from the mean on a Swedish growth chart [10].

The classification into aetiological periods was based on given clinical criteria, combined with available neuroimaging information [7]. Peri‐ and neonatal data, as well as CP type, were derived from medical and habilitation records. The findings at ultrasound, and magnetic resonance imaging (MRI) were classified into the following categories: maldevelopment, white matter lesions, cortical/subcortical lesions, basal ganglia/thalamus lesions, other findings and normal [11]. MRI findings were also classified according to the MRI Classification System of the SCPE (MRICS) [12]. Hypoxic‐ischaemic encephalopathy (HIE) was considered in children born after at least 34 weeks of gestation in the presence of two or more of the following symptoms/signs: Apgar score less than five at 5 min, resuscitation/assisted ventilation and convulsions before day 3 [13]. The following risk factors associated with CP were recorded: low Apgar score (less than five at 5 min), multiple birth, SGA and LGA, in the aetiologically unclassifiable group. The criteria of the American College of Obstetricians and Gynecologists (ACOG) [14] regarding intra‐partum events severe enough to cause CP were applied in children born after at least 34 weeks of gestation, that is, born near term (born after 34–36 weeks of gestation) or at term. The division into < 34 and ≥ 34 weeks of gestation was also applied when reporting MRI findings (Table 1). Population statistics, provided by the Medical Birth Registry Office at the Swedish National Board of Health and Welfare, were used to calculate prevalence of CP per 1000 live births, and per 1000 neonatal survivors, as well as perinatal and neonatal mortality.

**TABLE 1:** Neuroimaging findings on magnetic resonance imaging (MRI) by cerebral palsy (CP) type and gestational age group in 174 children born in 2015–2018.

MRI finding by CP type	Hemiplegia		Diplegia		Tetraplegia		Dyskinetic CP		Ataxia		Total, *n* (%)
	< 34 weeks	≥ 34 weeks	< 34 weeks	≥ 34 weeks	< 34 weeks	≥ 34 weeks	< 34 weeks	≥ 34 weeks	< 34 weeks	≥ 34 weeks	
Maldevelopment	1	5	0	5	0	1	0	1	0	0	13 (7.5)
White matter lesions	13	35	27	14	1	0	1	0	2	0	93 (53.4)
Cortical/subcortical lesions	0	25	0	2	0	2	0	3	0	0	32 (18.4)
Basal ganglia/thalamus lesions	0	0	0	2	0	4	0	12	0	1	19 (10.9)
Other abnormalities	0	5	2	0	1	0	0	0	0	0	8 (4.6)
Normal findings	2	2	2	1	0	0	0	2	0	0	9 (5.2)
Total	16	72	31	24	2	7	1	18	2	1	174 (100)
MRI not performed	1^a^	4	1	0	0	0	0	0	0	0	6

^a^ White matter lesion on neonatal ultrasound.

### 2.1 Birth‐Year Cohorts and Comparisons

The overall study included children born 1954–2018 for the purpose of reporting prevalence over time. Prevalence by gestational age group and by CP types was reported by 4‐year cohorts 1975–2018.

For the focus birth‐year cohort 2015–2018, new data on prevalence, neuroimaging, aetiology and motor function were reported. Comparisons were then made with the following birth‐year cohorts: 1999–2002 (*n* = 177), 2003–2006 (*n* = 195), 2007–2010 (*n* = 198) and 2011–2014 (*n* = 188) (post‐neonatal cases excluded). Access to neuroimaging results was over 90% in all included 4‐year cohorts.

Gross motor function was classified according to the Gross Motor Function Classification System (GMFCS) levels I–V, where children at level V require the most assistance [14]. GMFCS levels I–II represented independent walking ability, while levels IV–V corresponded to wheelchair mobility.

### 2.2 Ethics

The study was approved by the Regional Ethical Review Board in Göteborg, reference number 165‐15.

### 2.3 Statistical Methods

Crude mean prevalence was calculated as total number of CP cases in the 4‐year period divided by the population of the same birth years in the area and presented per 1000 live births. Percentages were calculated by total number of pre‐ or peri/neonatal cases. For comparisons between groups, the chi‐square test was used. For trends, the Cochran‐Armitage *χ* ^2^ test for trends was used. For comparison of independent samples, Kruskal–Wallis *H* was used. The level of significance was set at 0.05. Regression analysis was used to study crude prevalence by 4‐year cohort from 1999–2002 to 2015–2018.

## 3 Results

**FIGURE 1:**
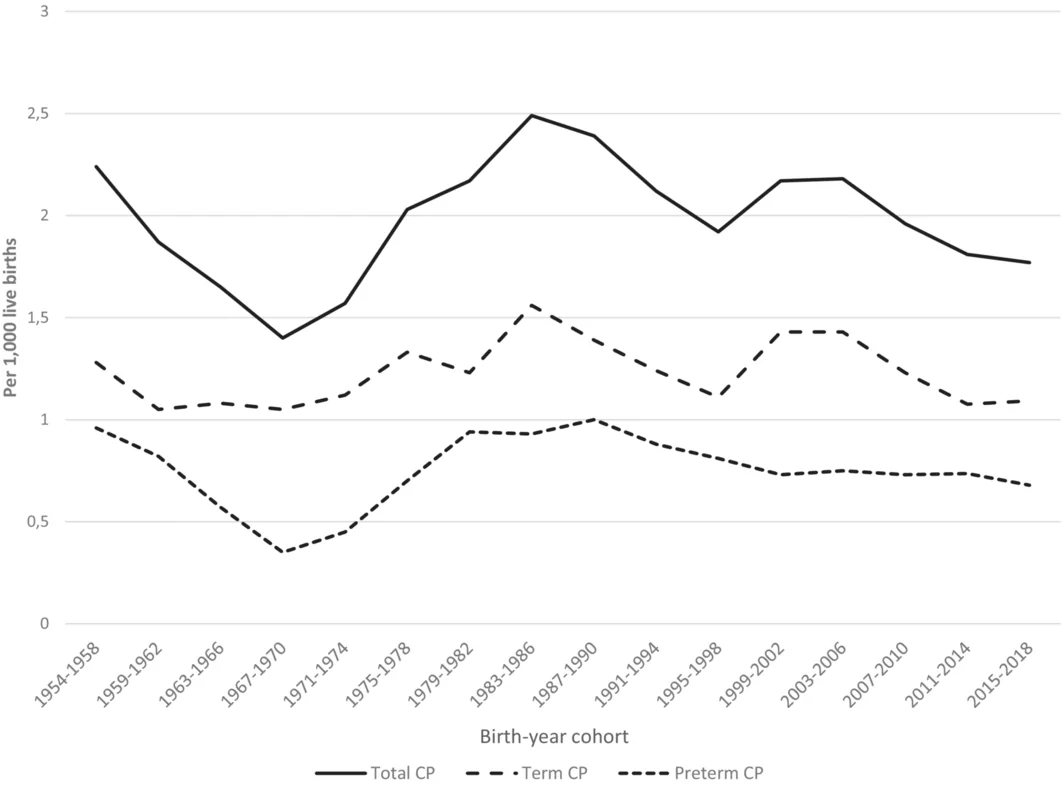
Crude prevalence of cerebral palsy (CP) per 1000 live births, in the birth‐year period 1954–2018 in western Sweden.

In the birth‐year period 2015–2018, 194 children with CP were identified in the study area. The crude mean prevalence was 1.78 per 1000 live births, 1.092 in children born at term and 0.688 in children born preterm (Figure 1). From the birth‐year cohort 1999–2002 to 2015–2018, a decrease in prevalence was observed (*p* = 0.01).

Fourteen children had an obvious post‐neonatal cause of their CP. They are excluded in the following and reported separately. The average perinatal mortality rate during the same period was 4.6 per 1000 births and the average neonatal mortality rate was 1.23 per 1000 live births. The neonatal mortality rate for children born extremely preterm was 161 per 1000 live births.

Of 180 children, 10.0% (18/180) were born extremely preterm, 13.9% (25/180) very preterm, 15.6% (28/180) moderately preterm and 60.6% (109/180) at term. Fifteen of the latter group were born post‐term.

A birth weight of < 1000 g was found in 10.0% (18/180), 8.3% (15/180) had a birth weight of 1000–1499 g, 22.8% (41/180) a birth weight of 1500–2499 g and 58.9% (106/180) a birth weight of 2500 g or more. Multiple births constituted 12.8% (23/180). All were twin pregnancies, resulting in 23 children with CP. In four of the twin pregnancies, twin to twin transfusion had occurred.

Eight children diagnosed with CP had died after the age of 2 years. SGA was found in 5.0% (9/180) and LGA in 8.9% (16/180).

Mean maternal age was 30.2 years compared with the average age of 30.4 years in the general population. The child with CP was the mother's first child in 53% (95/180), the second child in 28% (50/180) and child number three or more in 19% (35/180). Care in a neonatal intensive care unit had been given to 70% of the children (126/180) as compared with about 10% in the general population. Of the 126 in need of neonatal care, 51 were born at term and seven were post‐term.

There were 11 children with CP born after in vitro fertilization, eight singleton and three from twin pregnancies (6.1% compared with 6.4% in 2011–2014). Two children in this group were born at < 28 weeks, four at 28–31 weeks and five were born at term.

### 3.1 Gender Distribution

Of the 180 children, 60% (108/180) were boys and 72 were girls, a similar distribution to the previous cohort 2011–2014. In the term‐born group, 57 were boys and 52 were girls. In the children born preterm, 51 were boys and 20 girls.

### 3.2 Prevalence

**FIGURE 2:**
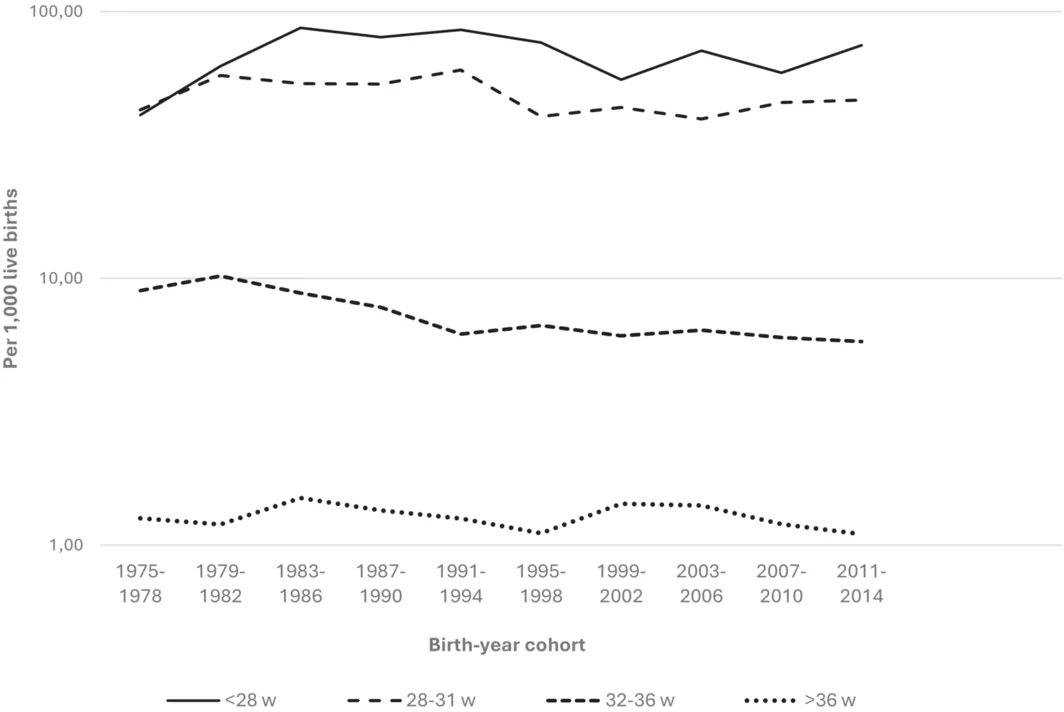
Prevalence per 1000 live births of cerebral palsy (CP) by gestational age group in the birth years 1975–2018 in western Sweden. Gestational age groups: < 28, 28–31, 32–36 and > 36 weeks of gestation.

Gestational age‐specific prevalence for < 28 gestational weeks was 65.9 per 1000 live births (18/273), 41.5 for 28–31 weeks (25/603), 5.6 for 32–36 weeks (28/5015) and 1.06 per 1000 for > 36 weeks of gestation (109/103072). The birth‐weight‐specific prevalence was 62 per 1000 for a birth weight of < 1000 g (18/290), 34 for a birth weight of 1000–1499 g (15/441), 11 per 1000 for a birth weight of 1500–2499 g (41/3712) and 1.0 per 1000 for a birth weight of 2500 g or more (106/104465). The prevalence by gestational age is shown in Figure 2. The prevalence of CP in children born before 28 weeks was 78.6 per 1000 neonatal survivors, compared with 87.1 per 1000 in the previous 4‐year cohort.

### 3.3 Predominant CP Types

**FIGURE 3:**
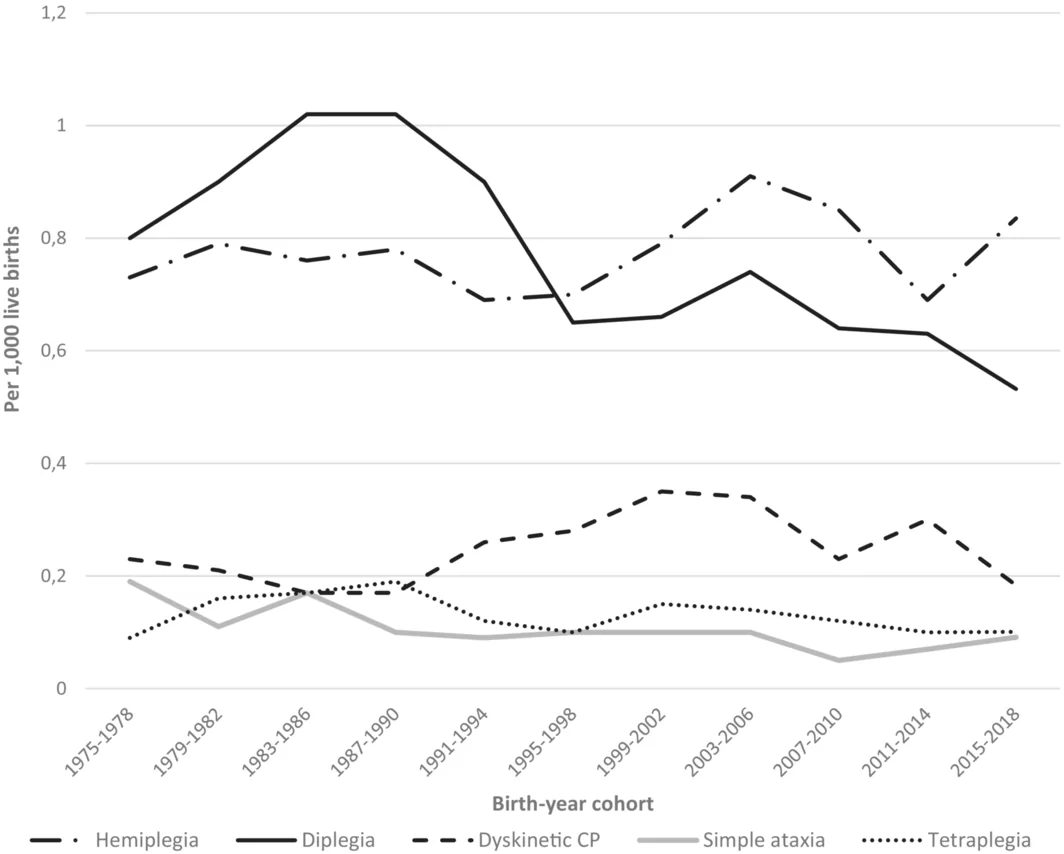
Prevalence of cerebral palsy (CP) by CP type, according to Hagberg's classification, per 1000 live births, in the birth‐year period 1975–2018.

**FIGURE 4:**
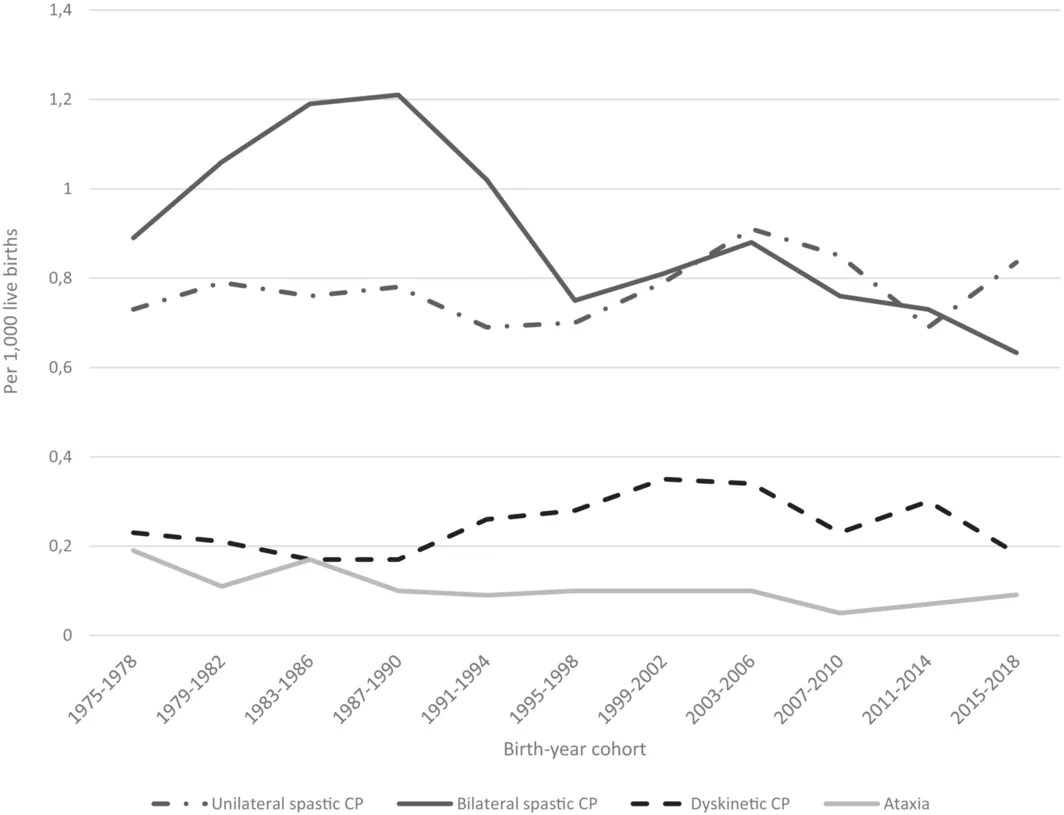
Prevalence of cerebral palsy (CP) by CP type, according to the classification by Surveillance of Cerebral Palsy in Europe (SCPE) per 1000 live births, in the birth‐year period 1975–2018.

The prevalence by CP type in the birth years 1975–2018 according to the Swedish classification is shown by 4‐year cohort in Figure 3, and according to the SCPE classification in Figure 4. Hemiplegia, or unilateral spastic CP, was found in 51.7% (93/180). Diplegia was found in 30.6% (55/180) and tetraplegia in 5.6% (10/180), together representing bilateral spastic CP, 36.1%.

**FIGURE 5:**
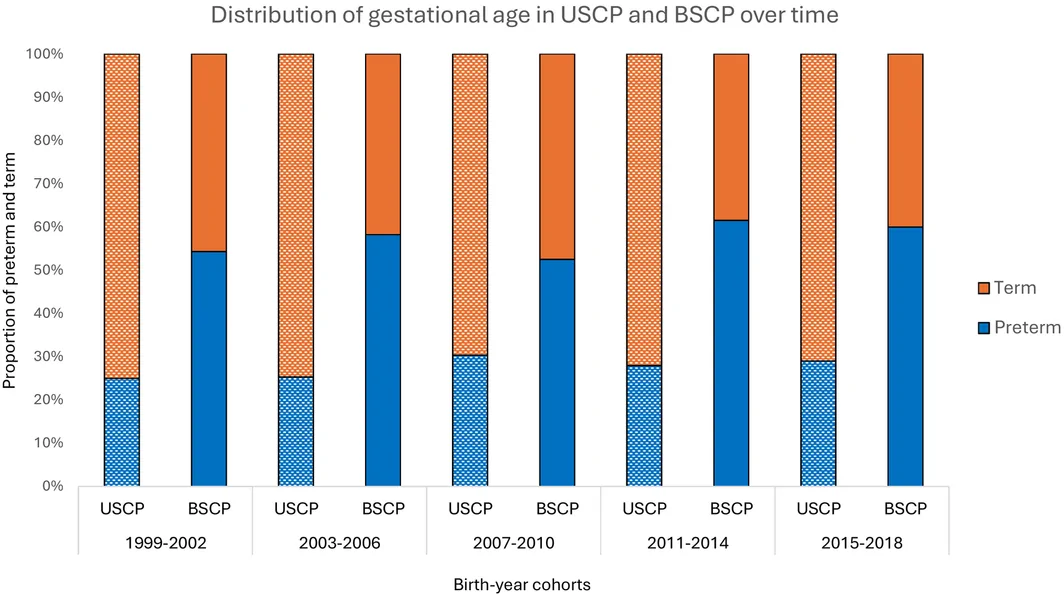
Distribution by gestational age group (preterm < 37 weeks, term > 36 weeks of gestation) in unilateral spastic cerebral palsy (USCP) and bilateral spastic cerebral palsy (BSCP) in the birth years 1999–2018.

Of the 65 children with bilateral spastic CP, 39 were born preterm. The bilateral spastic CP group has shown a larger proportion of preterm‐born children over the years, while in unilateral spastic CP the main proportion remains born at term (Figure 5).

Dyskinetic CP was found in 10.6% (19/180), showing a variable occurrence 1999–2018 (*χ* ^2^_trend_ = 1.84; ns). Ataxia was found in 1.7% (3/180).

### 3.4 Neuroimaging

Neuroimaging information was available in 97% (175/180). In one case only ultrasound was available, showing periventricular white matter lesion. The findings on MRI in 174 children are presented by CP type and gestational age group in Table 1. MRI revealed maldevelopment in 7%, white matter lesions in 54%, grey matter lesions in 29%, while normal findings were the case in 5% and miscellaneous findings in 5%. The proportion of white matter lesions in the total group had increased from the birth‐year cohort 1999–2002 to 2015–2018. This trend occurred both in the preterm and the term group (*χ* ^2^_trend_ = 10.41, *p* = 0.001, and *χ* ^2^_trend_ = 8.46, *p* = 0.004). In hemiplegia, or unilateral spastic CP, the background was white matter lesion in half, and middle cerebral artery infarction in 27%.

### 3.5 Aetiological Period

Distribution of aetiological period is presented in Table 2. In 34.4% (62/180), the background of CP was considered prenatal, and main causes were periventricular lesions, followed by cerebral maldevelopments.

**TABLE 2:** Aetiological periods by gestational age group in 180 children with cerebral palsy (CP) born in 2015–2018. Gestational age groups: < 28, 28–31, 32–36 and > 36 weeks of gestation.

Aetiological period	Gestational age				Total
	< 28 weeks	28–31 weeks	32–36 weeks	> 36 weeks	
**Prenatal**	1	1	10	50	62
Cerebral maldevelopment *or* other prenatal abnormalities	0	1	1	11	13
Intracranial haemorrhage/infarction/hydrocephalus	1	0	2	5	8
For children born at ≥ 34 weeks of gestation with normal delivery and peri/neonatal period: periventricular atrophy/porencephaly	0	0	7	34	41
**Perinatal/neonatal**	17	14	9	49	89
Intracranial haemorrhage/infarction/neonatal shock/brain oedema ± HIE *or* children born at ≥ 34 weeks of gestation: HIE	6	3	8	45	62
CNS infection *and/or* sepsis	1	2	0	4	7
For children born at < 34 weeks of gestation with periventricular atrophy *and/or* intracerebral haemorrhage with normal initial ultrasound	4	7	1		12
Probable perinatal/neonatal: for children born at < 34 weeks of gestation: low Apgar and/or low pH/mechanical ventilation > 7 days *or* complicated with pneumothorax	6	2	0		8
**Unclassifiable**	0	9	10	10	29
**Total**	18	24	29	109	180

In 49.4% (89/180), CP was considered having peri‐ or neonatal origin. For preterm children the main cause was cerebral haemorrhage in the peri‐ or neonatal period, while in term‐born children, cerebral infarctions or HIE were the main causes.

Among children born ≥ 34 weeks, 17 had sustained HIE, 10 of whom fulfilled the essential ACOG criteria of an intra‐partum hypoxic event severe enough to cause CP. In total, 10 children had undergone therapeutic hypothermia. Initial data were missing in two children born with asphyxia outside hospital, thus not classifiable according to ACOG. Both were diagnosed with dyskinetic CP.

### 3.6 Cases Unclassifiable as to Aetiological Period

In 29 children, aetiological criteria did not apply. Preterm birth had occurred in 21 cases, 11 of whom were born by emergency caesarean section. Complications during labour had occurred in eight cases (two cases of severe bleeding, one cord prolapse, five abnormal presentations). Three of 29 were from multiple birth, two were SGA and three were LGA. A maternal chronic disorder was found in nine cases. MRI revealed normal findings in five and other abnormalities in three, while seven had not undergone MRI. In 13 of 14 children born preterm with white matter lesions on later MRI, no early ultrasound was at hand, making it hard to date the lesions as prenatal or peri‐ or neonatal, respectively.

### 3.7 Motor Outcome—Comparison With Previous Cohorts

**FIGURE 6:**
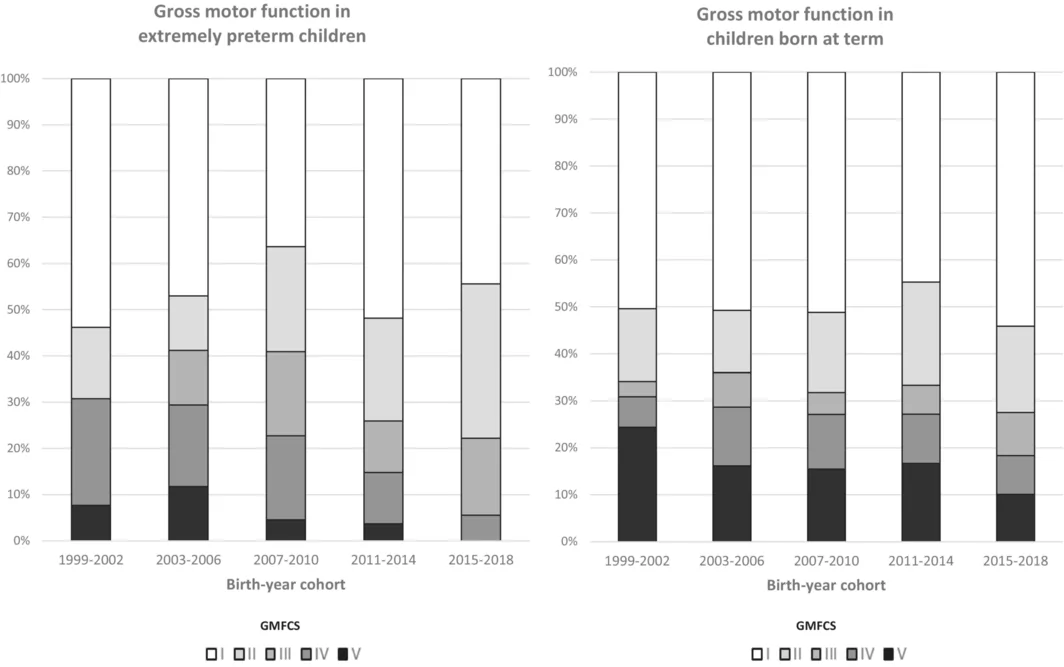
Gross motor function classification (GMFCS levels I–V) in children born extremely preterm, and children born at term, in the birth years 1999–2018.

In the total group of children born in 2015–2018, 72% walked independently (GMFCS I‐II), compared to 64% in the previous 4‐year cohort. A decrease of severe motor impairment (GMFCS IV‐V) was seen both in children born at term (*χ* ^2^_trend_ = 4.54, *p* = 0.03) and extremely preterm (*χ* ^2^_trend_ = 4.62, *p* = 0.03) from 1999 to 2018 (Figure 6).

### 3.8 Motor Function According to Aetiological Period

**FIGURE 7:**
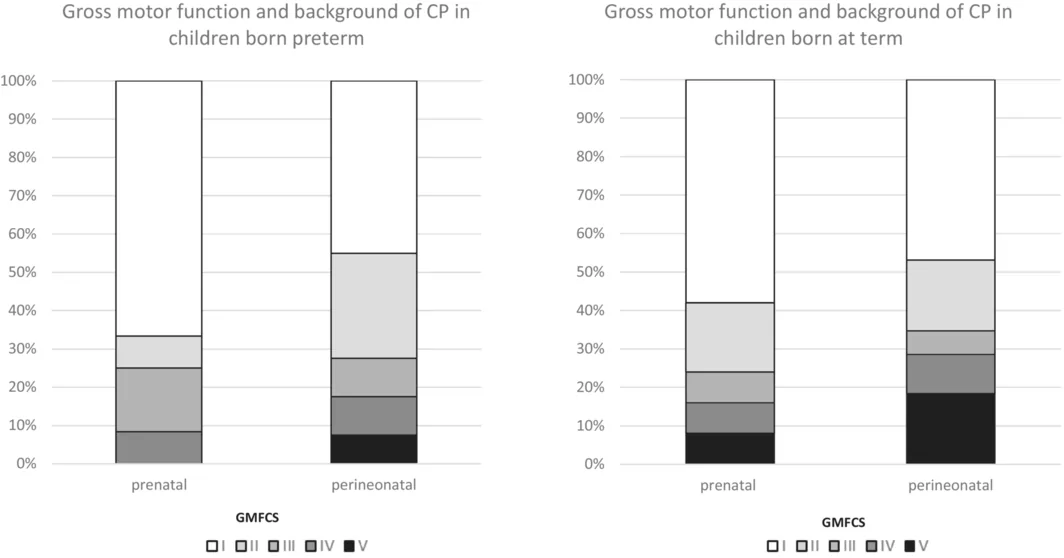
Gross motor function classification (GMFCS levels I–V) in children born 1999–2018 with prenatal and peri‐ or neonatal background of cerebral palsy, respectively.

There was no significant difference in distribution of GMFCS levels between prenatal, peri‐ or neonatal or unclassifiable aetiology in the children born 2015–2018 (*p* = 0.11). In children with a peri/neonatal background of CP there was a trend towards milder motor impairment over time, when comparing birth‐year cohorts 1999–2018. This was true both in preterm and term‐born children (*χ* ^2^_trend_ = 5.06, *p* = 0.02 for preterm, and *χ* ^2^_trend_ = 6.02, *p* = 0.01 for children born at term). No such trend was found in children with prenatal background of CP, nor for children where the given aetiological criteria were not applicable. The children with prenatal aetiology had a milder motor impairment than those with peri‐ or neonatal background of CP (*χ* ^2^ = 4.67, *p* = 0.04) (Figure 7).

### 3.9 Post‐Neonatal Cases

Among the 14 children with a post‐neonatal aetiology of CP born in 2015–2018, all but four were born at term. Hemiplegia was found in four children (three at GMFCS level I, one at level III), diplegia in three (GMFCS levels I–III), tetraplegia in four (all at GMFCS level V) and dyskinetic CP in three children (all at GMFCS level V). Five cases were due to trauma, three due to severe infection, while hydrocephalus, stroke and cardiac arrest (related to aspiration, sudden infant death episode, adverse pharmacological reaction and cardiac surgery respectively) accounted for one case each.

## 4 Discussion

During the last 20 years the CP prevalence has decreased from 2.18 to 1.78 per 1.000 live births in the study area [15]. Although risk factors and background of CP are better described and defined, motor presentation and classification of CP are still fundamental in CP research [16, 17]. Maintaining rigour in repeated follow‐up enables the study of changes over time, and increases the possibility to identify, prevent or reduce the effect of risk factors leading to CP.

Increased survival in extremely preterm, and children sustaining severe adverse events, creates challenges for healthcare. A Swiss study reported more bilateral forms and more children with severe CP following new guidelines regarding care for preterm‐born children at the limit of viability [18]. In the birth‐year cohort born in 2015–2018, the neonatal mortality in extremely preterm children had decreased, as well as the prevalence of CP by neonatal survivors. In a Danish study of birth years up to 2013, CP in very preterm (week 28–31) children had increased [19]. This was not the case in the present study; rather, a decrease was seen in this group.

We have earlier reported a decreasing severity of motor impairment in children with dyskinetic CP [3]. In the present study a trend towards milder gross motor impairment in the total CP group was found, both in children born at term and extremely preterm. CP after neonatal encephalopathy is still associated with a severe impairment [20]. We found an increase of children who had sustained HIE at more severe GMFCS levels than in the previous cohort. This proportion has varied over the 20‐year period studied, and no trend was detected. Peri‐ or neonatal background of CP was associated with a more severe motor impairment than prenatal background.

Unilateral and bilateral spastic CP, which constitute the largest groups, have varied over time. Of these groups, unilateral spastic CP showed an increase and dominates in the present study cohort. Dyskinetic CP has decreased both in percentage and motor severity.

The proportion of white matter lesions on MRI had increased over time, in both preterm and term‐born children, pointing towards brain compromise in early third trimester as the dominating background of CP in both gestational age groups. White matter lesions are mainly considered lesional, although genetics may play a role [21, 22].

Post‐neonatally acquired CP was more frequent in this birth‐year period than before. However, this is a small group of variable presence over time, requiring specific studies, which are underway. A classification of post‐neonatal causes has recently been developed by the SCPE, allowing a more detailed study of the background of post‐neonatal CP [23], will be employed in future studies from this register.

### 4.1 Future Perspectives

The children included in the present study, with a gestational age range of 22–42 weeks, were born before a more active management of pregnancies lasting more than 41 weeks was introduced in Sweden, with the aim to reduce mortality and adverse peri‐ or neonatal outcomes. It remains to be seen whether this change in management will affect the prevalence of CP [24]. Also, the introduction of magnesium sulphate to mothers in premature labour in 2020 as neuroprotection may affect the occurrence of CP in the offspring [25, 26]. The current guidelines regarding births at week 22 are under debate in Sweden. The Swedish National Council on Medical Ethics has recently published a statement on ethical aspects, recommending neonatal comfort care in births week 22 + 0 to 22 + 6 [27].

These examples underline the importance of monitoring the prevalence, aetiology and function in the field of cerebral palsy. As previously pointed out by Horber et al. [28] and Berry et al. [29], MRI findings may inform genetic investigation choices, shedding light on cases with unknown aetiology. Too few children in the study area are currently investigated to know the true contribution of genetics to the causal pathways leading to CP. These pathways are often complex but may be further elucidated using directed acyclic graphs [30].

## Author Contributions

**Kate Himmelmann:** conceptualization, methodology, data curation, investigation, validation, formal analysis, project administration, resources, writing – original draft, writing – review and editing, funding acquisition, visualization. **Magnus Påhlman:** methodology, data curation, investigation, writing – original draft, writing – review and editing, formal analysis.

## Funding

This study was supported by Swedish Government grants (ALFGBG‐1006593).

## Conflicts of Interest

The authors declare no conflicts of interest.

## Data Availability

Research data are not shared.

## References

[1] Statistikmyndigheten SCB (Statistics Sweden) , accessed April 24, 2026, https://www.scb.se/hitta‐statistik/sverige‐i‐siffror/manniskorna‐i‐sverige/befolkningstathet‐i‐sverige/.

[2] S. J. Hollung , T. Vik , S. Lydersen , I. J. Bakken , and G. L. Andersen , “Decreasing Prevalence and Severity of Cerebral Palsy in Norway Among Children Born 1999 to 2010 Concomitant With Improvements in Perinatal Health,” European Journal of Paediatric Neurology 22, no. 5 (2018): 814–821. 29779984 10.1016/j.ejpn.2018.05.001

[3] S. McIntyre , S. Goldsmith , A. Webb , et al., “Global Prevalence of Cerebral Palsy: A Systematic Analysis,” Developmental Medicine and Child Neurology 64, no. 12 (2022): 1494–1506. 35952356 10.1111/dmcn.15346 PMC9804547

[4] K. Himmelmann and M. Påhlman , “The Panorama of Cerebral Palsy in Sweden Part XIII Shows Declining Prevalence in Birth‐Years 2011–2014,” Acta Paediatrica 112, no. 1 (2023): 124–131. 36153696 10.1111/apa.16548 PMC10092185

[5] B. Hagberg , G. Hagberg , and I. Olow , “The Changing Panorama of Cerebral Palsy in Sweden 1954–1970. I. Analysis of the General Changes,” Acta Paediatrica Scandinavica 64, no. 2 (1975): 187–192. 1130174 10.1111/j.1651-2227.1975.tb03820.x

[6] P. Rosenbaum , N. Paneth , A. Leviton , et al., “A Report: The Definition and Classification of Cerebral Palsy April 2006,” Developmental Medicine and Child Neurology. Supplement 109 (2007): 8–14. 17370477

[7] B. Hagberg , G. Hagberg , I. Olow , and L. von Wendt , “The Changing Panorama of Cerebral Palsy in Sweden. VII. Prevalence and Origin in the Birth Year Period 1987–90,” Acta Paediatrica 85, no. 8 (1996): 954–960. 8863878 10.1111/j.1651-2227.1996.tb14193.x

[8] Surveillance of Cerebral Palsy in Europe , “Surveillance of Cerebral Palsy in Europe: A Collaboration of Cerebral Palsy Surveys and Registers,” Developmental Medicine and Child Neurology 42, no. 12 (2000): 816–824. 11132255 10.1017/s0012162200001511

[9] P. Uvebrant , “Hemiplegic Cerebral Palsy. Aetiology and Outcome,” Acta Paediatrica Scandinavica. Supplement 345 (1988): 1–100. 3201989 10.1111/j.1651-2227.1988.tb14939.x

[10] A. Niklasson , A. Ericson , J. G. Fryer , J. Karlberg , C. Lawrence , and P. Karlberg , “An Update of the Swedish Reference Standards for Weight, Length and Head Circumference at Birth for Given Gestational Age (1977–1981),” Acta Paediatrica Scandinavica 80, no. 8–9 (1991): 756–762. 1957592 10.1111/j.1651-2227.1991.tb11945.x

[11] I. Krägeloh‐Mann , “Imaging of Early Brain Injury and Cortical Plasticity,” Experimental Neurology 190, no. S1 (2004): 84–90. 10.1016/j.expneurol.2004.05.037 15498546

[12] K. Himmelmann , V. Horber , J. De La Cruz , et al., “MRI Classification System (MRICS) for Children With Cerebral Palsy: Development, Reliability, and Recommendations,” Developmental Medicine and Child Neurology 59, no. 1 (2017): 57–64. 27325153 10.1111/dmcn.13166

[13] I. Krägeloh‐Mann , G. Hagberg , C. Meisner , et al., “Bilateral Spastic Cerebral Palsy—A Collaborative Study Between Southwest Ern Germany and Western Sweden. III; Aetiology,” Developmental Medicine and Child Neurology 37, no. 3 (1995): 191–203. 7890124 10.1111/j.1469-8749.1995.tb11992.x

[14] ACOG: The American College of Obstetricians and Gynecologists' Task Force on Neonatal Encephalopathy and Cerebral Palsy , The American College of Obstetricians and Gynecologists , and The American Academy of Pediatrics , Neonatal Encephalopathy and Cerebral Palsy: Defining the Pathogenesis and Pathophysiology (American College of Obstetricians and Gynecologists, 2003).

[15] R. Palisano , P. Rosenbaum , S. Walter , D. Russell , E. Wood , and B. Galuppi , “Development and Reliability of a System to Classify Gross Motor Function in Children With Cerebral Palsy,” Developmental Medicine and Child Neurology 39, no. 4 (1997): 214–223. 9183258 10.1111/j.1469-8749.1997.tb07414.x

[16] K. Himmelmann , G. Hagberg , and P. Uvebrant , “The Changing Panorama of Cerebral Palsy in Sweden. X. Prevalence and Origin in the Birth‐Year Period 1999–2002,” Acta Paediatrica 99, no. 9 (2010): 1337–1343. 20377538 10.1111/j.1651-2227.2010.01819.x

[17] I. Krägeloh‐Mann , J. de la Cruz , M. Delobel‐Ayoub , et al., “The Distinction Between the Definition and Description of Cerebral Palsy,” Developmental Medicine and Child Neurology 68, no. 4 (2026): 579–580. 40722232 10.1111/dmcn.16422

[18] E. Monbaliu , S. Bekteshi , J. Vermeulen , et al., “Motor Classification Remains Essential in Describing Cerebral Palsy,” Developmental Medicine and Child Neurology 68, no. 4 (2026): 591–592. 40717283 10.1111/dmcn.16430

[19] L. Quinten , G. Natalucci , M. Adams , et al., “Incidence, Subtypes and Severity of Cerebral Palsy in Infants Born Extremely Preterm in Switzerland: A Retrospective Study Comparing Two Time Periods,” Early Human Development 209 (2025): 106328. 40609377 10.1016/j.earlhumdev.2025.106328

[20] M. V. Fogh , G. Greisen , T. D. Clausen , L. Krebs , M. L. Larsen , and C. E. Hoei‐Hansen , “Increasing Prevalence of Cerebral Palsy in Children Born Very Preterm in Denmark,” Developmental Medicine and Child Neurology 67, no. 1 (2025): 68–76. 38994777 10.1111/dmcn.16020 PMC11625462

[21] M. R. Battin , A. Mulqueeney , A. Mackey , A. Sorhage , W. Alzaher , and N. Stott , “Cerebral Palsy Associated With Neonatal Encephalopathy: Review of Te Rēhita a Hōkai Nukurangi Aotearoa‐The New Zealand Cerebral Palsy Register (NZCPR) Data,” Journal of Paediatrics and Child Health 61, no. 11 (2025): 1788–1794. 40899614 10.1111/jpc.70186

[22] V. Horber , U. Grasshoff , E. Sellier , C. Arnaud , I. Krägeloh‐Mann , and K. Himmelmann , “The Role of Neuroimaging and Genetic Analysis in the Diagnosis of Children With Cerebral Palsy,” Frontiers in Neurology 11 (2021): 628075. 33633660 10.3389/fneur.2020.628075 PMC7900404

[23] L. Pudig , M. Delobel‐Ayoub , K. Horridge , et al., “Classification of Events Contributing to Postneonatal Cerebral Palsy: Development, Reliability, and Recommendations for Use,” Developmental Medicine and Child Neurology 66, no. 2 (2024): 250–257. 37488719 10.1111/dmcn.15710

[24] K. Källén , M. Norman , C. Elvander , et al., “Maternal and Perinatal Outcomes After Implementation of a More Active Management in Late‐ and Postterm Pregnancies in Sweden: A Population‐Based Cohort Study,” PLoS Medicine 22, no. 1 (2025): e1004504. 39820829 10.1371/journal.pmed.1004504 PMC11737695

[25] S. Hellström , A. Jonsdotter , M. Jonsson , et al., “A Follow Up on the Feasibility After National Implementation of Magnesium Sulfate for Neuroprotection Prior to Preterm Birth,” Acta Obstetricia et Gynecologica Scandinavica 102, no. 12 (2023): 1741–1748. 37680134 10.1111/aogs.14673 PMC10619608

[26] G. Koning , A. L. Leverin , S. Nair , et al., “Magnesium Induces Preconditioning of the Neonatal Brain via Profound Mitochondrial Protection,” Journal of Cerebral Blood Flow and Metabolism 39, no. 6 (2019): 1038–1055. 29206066 10.1177/0271678X17746132 PMC6547197

[27] Smer—Statens medicinsk‐etiska råd (Swedish National Council on Medical Ethics) , “Vård och behandling av extremt för tidigt födda barn—etiska aspekter (2026:3),” https://smer.se/wp‐content/uploads/2026/03/yttrande‐vard‐och‐behandling‐av‐extremt‐for‐tidigt‐fodda‐barn.pdf.

[28] V. Horber , E. Sellier , K. Horridge , et al., “The Origin of the Cerebral Palsies: Contribution of Population‐Based Neuroimaging Data,” Neuropediatrics 51, no. 2 (2020): 113–119. 32120429 10.1055/s-0039-3402007

[29] J. G. Berry , A. Taranath , R. Goetti , et al., “Genetic Diagnostic Yield by MRI Pattern in Children With Cerebral Palsy: A Population‐Based Study,” eBioMedicine 122 (2025): 106013. 41202470 10.1016/j.ebiom.2025.106013 PMC12790587

[30] S. Goldsmith , S. McIntyre , N. Badawi , et al., “The Cerebral Palsy Directed Acyclic Graph: A Structural Causal Model of Aetiology,” Developmental Medicine and Child Neurology, ahead of print, April 9, (2026), 10.1111/dmcn.70267. PMC13649292 41955499

